# Supplementary material for: Tuning the open-close equilibrium of Cereblon with small molecules influences protein degradation
Source: bioRxiv. 2025 Dec 22:2025.12.19.695617. Preprint. [Version 1] doi: 10.64898/2025.12.19.695617 (PMC12767645; doi:10.64898/2025.12.19.695617)
Supplement: Supplement 1 [file media-1.pdf]

Supplementary Information

Tuning the open-close equilibrium of cereblon with small molecules influences protein degradation

Suzanne O'Connor[1,‡], *Zoe J. Rutter*[1,‡,Ⱦ], *Angus D. Cowan*[1], *Markus Zeeb*[2], *Florian Binder*[2], *Yuting Cao*[1], *Sohini Chakraborti*[1,ϕ], *Stefan Djukic*[1], *Leonhard Geist*[3], *Elizabeth Hogg*[1], *Giorgia Kidd*[1], *Matthias Krumb*[3,ˀ], *Simon Langer*[2], *Denis Schmidt*[2], *Liam Martin*[1], *Elisha H. McCrory*[1], *Giacomo Padroni*[1,¶], *Ilaria Puoti*[1], *Luke Simpson*[1,^], *Manon Sturbaut*[1], *Lisa Crozier*[1], *Vesna Vetma*[1,§], *Qingzhi Zhang*[1], *Kirsten McAulay*[1,*], *Theodor Theis*[2,*], *Alessio Ciulli*[1,*]

[1]Centre for Targeted Protein Degradation, School of Life Sciences, University of Dundee, Dundee, DD1 5JJ, UK.

[2]Boehringer Ingelheim Pharma GmbH & Co KG, Biberach an der Riß, Germany

[3]Boehringer Ingelheim RCV GmbH & Co KG, 1221 Vienna, Austria

*Corresponding Authors

Chemical Synthesis & Reagents 4
General Information 4
Analytical MS Methods and instrumentation 4
Experimental Procedures 5
Data Tables 12
Table S1. SPR Data Table CRBN^{midi} 12
Table S2. ITC measurements of CRBN binders with CRBN:DDB1$^{\Delta BPB}$ 12
Table S3. Measurement of different CRBN classes by DSF with CRBN^{midi}. Turbidity first derivative of thermal denaturation for CRBN^{midi} in the absence or presence of binders. .. 13
Table S4 - Turbidity first derivative of thermal denaturation for CRBN^{midi} WT and mutants in the absence or presence of binders. WT is mean of triplicates. 13
Table S5. Small Angle X-ray scattering statistics for CRBN^{midi} bound to each binder series 14
Table S6. Small Angle X-ray Scattering statistics for CRBN^{midi} mutants bound to compound 9 16
Table S7. X-Ray Crystallographic Data and Refinement Statistics Data 17
Table S8. Cryo-EM data collection, processing and refinement statistics for dihydrouracil **9** 18
Table S9. Cryo-EM data collection, processing and refinement statistics for 5,4-spiro **8** . 19
Table S10. List of primers for sire-directed mutagenesis 20
Table S11. List of cycling parameters for site-directed mutagenesis 20
Table S12. Intact protein LC-MS of CRBN^{midi} mutants 20
Table S13. Primers for cloning CRBN:DDB1$^{\Delta BPB}$ 20
Supplementary Figures 21
Figure S1. 2D ^{15}N HSQC CSP detected with thalidomide and 15N-labelled CRBN^{TBD} 21
Figure S2. CRBN^{midi}:Dihydrouracil **9** crystal structure domain swap and comparison to other structures. 21
Figure S3. Cryo-EM Workflow for dihydrouracil **9** 22
Figure S4. Cryo-EM Map Information for dihydrouracil **9** 23
Figure S5. Cryo-EM Workflow for 5,4-spiro **8** 24
Figure S6. Cryo-EM Map Information for 5,4-spiro **8** 25
Figure S7. 5,4-spiro **8** Binding Site in Locally Refined Cryo-EM Map 25

Figure S8. Validating the stability of FLAG-CRBN expressed into DLD-1 CRBN KO background cells. 26
Figure S9. BRD4 Degradation in DLD-1 WT, CRBN KO and CRBN KO stably expressing FLAG-CRBN upon Treatment with dBET6 26
References 27

Chemical Synthesis & Reagents

General Information

Commercially available dry solvents were obtained from Sigma Aldrich. All reagents, unless otherwise noted, were commercially available and purchased from Sigma Aldrich, Combi Blocks, ABCR, Fluorochem, Activate or Enamine, at least 95% pure and used without further purification. All reactions were carried out under a nitrogen atmosphere. Normal phase TLC was carried out on pre-coated silica plates (Kieselgel 60 F254, BDH) with visualization via UV light (UV 254 and/or 365 nm) and/or basic potassium permanganate solution. Isolute® phase separator columns from Biotage were used. Flash column chromatography was performed using either a Teledyne Isco Combiflash Rf or Rf200i, or a Biotage Isolera One with prepacked Redisep RF normal phase disposable columns. Reverse phase chromatography was carried out using Biotage SNAP-C18 columns or RediSep Rf Reversed Phase C18 Columns. Strong cation exchange (SCX) chromatography was carried out using Biotage Isolute SCX-2 columns. NMR Spectra were recorded on Bruker 400 MHz or 500 MHz spectrometers as specified. Chemical shifts are quoted in ppm and referenced to the residual solvent signals: ^{1}H NMR δ (ppm) = 7.26 ($CDCl_3$-d), 5.32 (CD_2Cl_2) or 3.31 (MeOD-d_4). Signal splitting patterns are described as singlet (s), doublet (d), triplet (t), quartet (q), quintet (quin.), multiplet (m), broad (br) or a combination thereof. Coupling constants (J) are measured in Hertz (Hz). Diastereomeric ratios (dr) were calculated using the ratios of NMR integrals. Chiral SFC was carried out by Reach Separations Ltd., using a Sepiatec SFC systems equipped with a Lux A1 column (21.2 mm x 250 mm, 5 um particle size). Samples were eluted with an isocratic gradient of 40:60 EtOH:CO_2 (0.2% v/v NH_3) over 10 min at a flow rate of 50 mL/min with an oven temperature of 40 °C. Diastereomeric mixture was dissolved in EtOH at 6 mg/mL and injected 16 times at 500 uL.

Abbreviations

aq. for aqueous, Boc for *N*-*tert*butyloxycarbonyl, DCM for dichloromethane, DMA for *N*,*N*-dimethylacetamide, DMF for *N*,*N*-dimethylformamide, DIPEA for N,N-diisopropylethylamine, DMSO for dimethylsulfoxide, HATU for 1-[bis(dimethylamino)methylene]-1*H*-1,2,3-triazolo[4,5-b]pyridinium 3-oxide hexafluorophosphate, TBAF for tetra-*N*-butylammonium fluoride

Analytical MS Methods and instrumentation

LCMS

Liquid chromatography-mass spectrometry (LCMS) was carried out on a Shimadzu HPLC/MS 2020 equipped with a Hypersil Gold column (1.9 µm particle size, 50 × 2.1 mm), photodiode array detector and ESI detector. Samples were eluted with either a 3 min or 5 min gradient of 5–95% acetonitrile:water containing 0.1% formic acid at a flow rate of 0.7 mL/min.

HPLC

Preparative HPLC was performed on a Waters Prep 150 LC system with a Waters XBridge C18 column (100 mm x 19 mm; 5 µm particle size) and a gradient of 5% to 95% acetonitrile in water over 20 minutes and a flow rate of 25 mL/min, with ammonia in the aqueous phase.

Experimental Procedures

Compound **1** is commercially available (CAS: 443955-72-4) and was purchased from Enamine.

Synthesis of compounds **9**, **10**, **11** and **12** were conducted according to literature references. [1–4]

Synthesis of 7-(4-Methylpiperazine-1-carbonyl)-1H,2H,3H-pyrido[2,3-b][1,4]oxazin-2-one (compound 2)

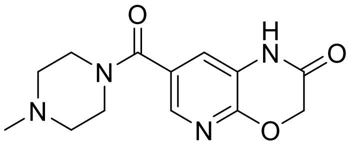

To a solution of 2-oxo-1H,2H,3H-pyrido[2,3-b][1,4]oxazine-7-carboxylic acid (200 µmol, 39.6 mg) in dimethylacetamide (1 mL), N,N-diisopropylethylamine (1160 µmol, 0.151 g, 0.2 mL) was added. The mixture was stirred briefly to ensure dissolution. Subsequently, HATU (210 µmol, 80 mg) was added, and the reaction mixture was stirred briefly to activate the acid. Once the activation was complete, 1-methylpiperazine (300 µmol, 0.031 g, 0.034 mL) was added to the reaction mixture. The resulting solution was stirred at room temperature for 3.5 hours. The progress of the reaction was monitored by LCMS, which confirmed complete conversion of the starting material. The reaction mixture was diluted with acetonitrile-water and subjected to purification using preparative HPLC. The purified product, 7-(4-methylpiperazine-1-carbonyl)-1H,2H,3H-pyrido[2,3-b][1,4]oxazin-2-one, was obtained as a solid (18.1 mg, 33% yield).

MS (ESI+): m/z calcd. for $[M+H^+]$: 277.12, found: 277.19.

^1H NMR (400 MHz, DMSO-d6) δ ppm: 10.91 (s, 1H), 7.83 (d, J = 2.0 Hz, 1H), 7.22 (d, J = 2.0 Hz, 1H), 4.83 (s, 2H), 3.36 - 3.78 (m, 4H), 2.26 - 2.43 (m, 4H), 2.21 (s, 3H).

Synthesis of 7-(1-methyl-1H-1,3-benzodiazol-2-yl)-1H,2H,3H-pyrido[2,3-b][1,4]oxazin-2-one (compound 3)

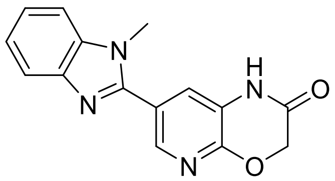

To a solution of 2-oxo-1H,2H,3H-pyrido[2,3-b][1,4]oxazine-7-carboxylic acid (100 µmol, 19.8 mg) in dimethylformamide (2 mL), N,N-diisopropylethylamine (0.3 mmol, 51.55 µL) was added at room temperature. The mixture was stirred briefly to ensure dissolution. Subsequently, HATU (0.11 mmol, 41.8 mg) was added, and the reaction mixture was stirred for 10 minutes to activate the acid. N-methyl-ortho-phenylenediamine (0.1 mmol, 12.2 mg) was then added to the reaction mixture. The resulting solution was stirred at room temperature for 1 hour. The progress of the reaction was monitored by LCMS, which confirmed the formation of the intermediate product. The reaction mixture was filtered through basic alumina and washed with a DMF/methanol mixture (9:1). The filtrate was concentrated under reduced pressure to yield a residue which was treated with acetic acid (2 mL) and refluxed. LCMS analysis confirmed

the formation of the desired product. The crude product was concentrated under reduced pressure, dissolved in dimethylformamide, and purified using reverse-phase liquid chromatography on a C18 column with an acetonitrile-water-trifluoroacetic acid (ACN/H2O/TFA) mobile phase at 50°C. The collected fractions were freeze-dried to yield the purified product as a yellow solid (28.9 mg, 73% yield).

MS (ESI+): m/z calcd. for [M+H$^+$]: 281.10, found: 281.19.

^{1}H NMR (400 MHz, DMSO-d6) δ ppm: 11.16 (s, 1H), 8.32 (d, *J* = 2.15 Hz, 1H), 7.85 (d, *J* = 7.86 Hz, 1H), 7.79 (d, *J* = 7.35 Hz, 1H), 7.70 (d, *J* = 2.15 Hz, 1H), 7.43 - 7.56 (m, 2H), 4.94 (s, 2H), 1.96 (s, 3H).

Synthesis of 7-(1-cyclohexyl-1H-1,3-benzodiazol-2-yl)-1H,2H,3H-pyrido[2,3-b][1,4]oxazin-2-one (compound 4)

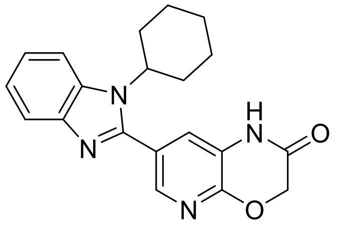

To a solution of 2-oxo-1H,2H,3H-pyrido[2,3-b][1,4]oxazine-7-carboxylic acid (100 µmol, 19.8 mg) in dimethylformamide (2 mL) at room temperature, N,N-diisopropylethylamine (0.3 mmol, 52 µL) was added. The mixture was stirred briefly to ensure dissolution. Subsequently, HATU (0.11 mmol, 41.8 mg) was added, and the reaction mixture was stirred for 10 minutes. N1-cyclohexylbenzene-1,2-diamine (0.1 mmol, 19.03 mg) was then added to the reaction mixture, and the resulting solution was stirred at room temperature for 1 hour. The progress of the reaction was monitored by LCMS, which confirmed the formation of the intermediate product. The reaction mixture was filtered through basic alumina and washed with a DMF/methanol mixture (9:1). The filtrate was concentrated under reduced pressure to yield crude residue. To induce cyclization, the residue was treated with acetic acid (2 mL) and refluxed. LCMS analysis confirmed the formation of the desired product. The crude product was concentrated under reduced pressure, dissolved in dimethylformamide, and purified using reverse-phase liquid chromatography on a C18 column with an acetonitrile-water-trifluoroacetic acid (ACN/H2O/TFA) mobile phase at 50°C. The purified product was obtained as a beige amorphous solid (30.4 mg, 87% yield).

MS (ESI+): m/z calcd. for [M+H$^+$]: 349.16, found: 349.21.

^{1}H NMR (400 MHz, DMSO-d6) δ ppm: 11.18 (s, 1H), 8.16 (s, 1H), 8.11 (dd, *J* = 5.7, 3.0 Hz, 1H), 7.80 (dd, *J* = 6.0, 3.1 Hz, 1H), 7.57 (s, 1H), 7.47 (dd, *J* = 6.0, 3.0 Hz, 2H), 5.14 (s, 1H), 4.95 (s, 3H), 4.22-4.45 (m, 2H), 2.18-2.43 (m, 4H), 2.00 (d, *J* = 11.3 Hz, 2H), 1.86 (d, *J* = 11.2 Hz, 3H), 1.65 (s, 1H), 1.30-1.55 (m, 4H).

Synthesis of 7-Pyridazin-3-yl-1,7-diazaspiro[4.4]nonan-2-one (compound 6)

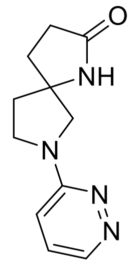

To a 5 mL microwave vial, 3-bromopyridazine (1.0 eq, 0.06 g, 0.378 mmol) and 1,7-diazaspiro[4.4]nonan-2-one (1.2 eq, 0.063 g, 0.452 mmol) were dissolved in N-methylpyrrolidone (2 mL) at room temperature. The resulting solution was degassed with nitrogen. DIPEA (0.415 eq, 0.0106 mL, 0.157 mmol) and potassium iodide (0.100 eq, 0.006 g, 0.0378 mmol) were then added. The reaction mixture was stirred at 170°C for 1.5 hours. The progress of the reaction was monitored by TLC and LCMS, which confirmed the complete consumption of the starting material and the formation of the desired product. The reaction mixture was diluted with ice water (20 mL) and extracted with ethyl acetate (2 × 10 mL). The aqueous layer was lyophilized to yield a crude material which was purified using preparative HPLC to yield the desired product (60 mg, 22%).

MS (ESI+): m/z calcd. for $[M+H^+]$: 219.12, found: 219.16.

^1H NMR (400 MHz, DMSO-d6) δ ppm: 2.06 (m, 4H), 2.29 (m, 2H), 3.43 (m, 1H), 3.51 (m, 2H), 3.58 (m, 1H), 6.83 (dd, J = 9.2, 1.2 Hz, 1H), 7.34 (dd, J = 9.2, 4.4 Hz, 1H), 8.12 (s, 1H), 8.48 (dd, J = 4.4, 1.2 Hz, 1H).

Synthesis of 2-Pyridazin-3-yl-2,5-diazaspiro[3.4]octan-6-one (compound 7)

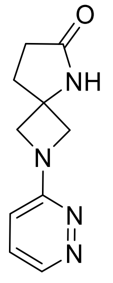

In a 5 mL microwave vial, 3-bromopyridazine (1.0 eq, 0.030 g, 0.189 mmol) and 2,5-diazaspiro[3.4]octan-6-one (1.5 eq, 0.034 g, 0.283 mmol) were dissolved in N-methylpyrrolidone (2 mL) at room temperature. DIPEA (2.00 eq, 0.065 mL, 0.378 mmol) and potassium iodide (0.100 eq, 0.003 g, 0.0189 mmol) were then added. The reaction mixture was stirred at 170°C for 1.5 hours. The progress of the reaction was monitored by TLC and LCMS, which confirmed the formation of the desired product, although the starting material was not completely consumed. The reaction mixture was diluted with ice water (20 mL) and extracted with ethyl acetate (2 × 10 mL). The aqueous layer was lyophilized to yield a crude compound (0.03 g). The crude product was purified using preparative HPLC to give the final product (30 mg, 70%).

MS (ESI+): m/z calcd. for $[M+H^+]$: 205.10, found: 205.15.

^{1}H NMR (400 MHz, DMSO-d6) δ ppm: 2.35 (m, 4H), 4.08 (dd, J = 31.6, 8.8 Hz, 4H), 6.83 (dd, J = 9.0, 1.6 Hz, 1H), 7.38 (dd, J = 9.0, 4.8 Hz, 1H), 8.28 (s, 1H), 8.57 (dd, J = 4.6, 1.6 Hz, 1H).

Synthesis of 2-(5-chloro-1-(piperidin-4-yl)-1H-indol-4-yl)-2,5-diazaspiro[3.4]octan-6-one (compound 8)

4-(4-bromo-5-chloro-indol-1-yl)piperidine-1-carboxylate

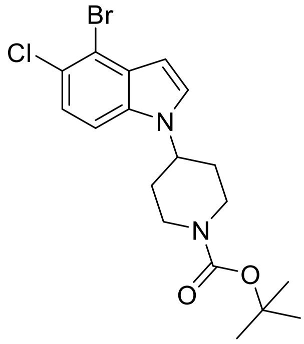

4-bromo-5-chloro-1H-indole (1 eq, 450 mg, 1.95 mmol), tert-butyl 4-methylsulfonyloxypiperidine-1-carboxylate (2 eq, 1091 mg, 3.90 mmol) and Cs_2CO_3 (3 eq, 1908 mg, 5.86 mmol) were dissolved in MeCN (15mL) and stirred at reflux under N_2 atmosphere for 18 h. A further 500 mg of mesylate was added and the reaction was stirred for 6 h, before the addition of another 500 mg. The reaction mixture was then stirred overnight. The solvent was removed from the reaction mixture and the crude material was taken up in EtOAc (30 mL) and water (30 mL). The organic layer was washed with water (3 x 30 ml) and then brine (30 ml) before being dried over anhydrous Na_2SO_4. The crude material was purified by flash reverse phase chromatography, elution 10 to 95% ACN in water + 0.1% formic acid, to afford the product tert-butyl 4-(4-bromo-5-chloro-indol-1-yl)piperidine-1-carboxylate (375 mg, 44%) as a colourless oil.

LCMS: m/z calcd. for $[M+H^+]$ 413.7, found: 412.9; RT = 2.24 min.

^{1}H NMR (500 MHz, $CDCl_3$) δ ppm: 7.26 (d, J = 4.2 Hz, 2H), 7.25 (d, J = 3.3 Hz, 1H), 6.58 (d, J = 3.3 Hz, 1H), 4.48 – 4.15 (m, J = 71.8, 67.9, 33.9 Hz, 3H), 2.92 (s, 2H), 2.14 – 2.01 (m, 2H), 1.90 (qd, J = 12.4, 4.2 Hz, 2H), 1.50 (s, 9H).

tert-butyl 4-[5-chloro-4-(6-oxo-2,5-diazaspiro[3.4]octan-2-yl)indol-1-yl]piperidine-1-carboxylate

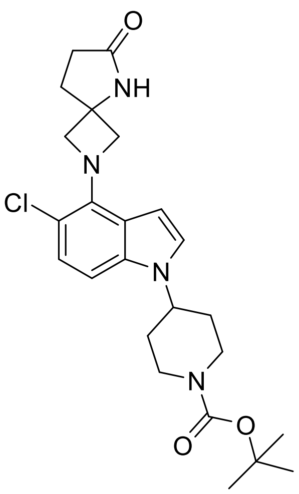

tert-butyl 4-(4-bromo-5-chloro-indol-1-yl)piperidine-1-carboxylate (300 mg, 0.723 mmol), 5-aza-2-azoniaspiro[3.4]octan-6-one;2,2,2-trifluoroacetate (208 mg, 0.868 mmol), RuPhos Pd G3 (60 mg, 0.0723 mmol) and Cs_2CO_3 (471 mg, 1.45 mmol) were dissolved in anhydrous dioxane (10mL) under an N_2 atmosphere. The reaction mixture was stirred at 100 °C for 18 h. The solvent was removed from the reaction mixture under reduced pressure and the crude material was purified by flash column chromatography (0-20% MeOH in CH_2Cl_2) to afford the product tert-butyl 4-[5-chloro-4-(6-oxo-2,5-diazaspiro[3.4]octan-2-yl)indol-1-yl]piperidine-1-carboxylate (65 mg, 17.6%) as a colourless solid.

LCMS: m/z calcd. for $[M+H^+]$ 459.2, found: 459.0; RT = 1.81 min.

^1H NMR (500 MHz, $CDCl_3$) δ ppm: 7.06 – 7.00 (m, 2H), 6.75 (d, J = 8.7 Hz, 1H), 6.55 (d, J = 3.3 Hz, 1H), 4.58 (d, J = 8.6 Hz, 2H), 4.46 (d, J = 8.6 Hz, 2H), 4.39 – 4.18 (m, 3H), 2.95 – 2.82 (m, 2H), 2.51 – 2.38 (m, 4H), 2.04 (t, J = 6.0 Hz, 2H), 1.84 (app qd, J = 12.4, 4.2 Hz, 2H), 1.49 (s, 9H).

2-(5-chloro-1-(piperidin-4-yl)-1H-indol-4-yl)-2,5-diazaspiro[3.4]octan-6-one (compound **8**)

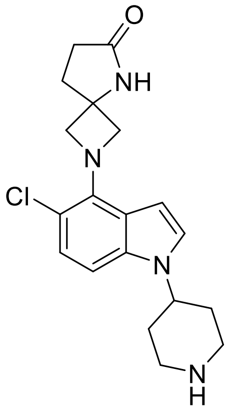

To a solution of tert-butyl 4-[5-chloro-4-(6-oxo-2,5-diazaspiro[3.4]octan-2-yl)indol-1-yl]piperidine-1-carboxylate (1.00 eq, 23 mg, 0.0401 mmol) in DCM (1mL) was added 2,2,2-trifluoroacetic acid (10.0 eq, 0.031 mL, 0.401 mmol). The solution was stirred at rt for 3h. After completeness of the reaction, the solvent was removed under reduced pressure. The crude was purified by preparative HPLC (Waters XSelect CSH C18 ODB column, 5μ silica, 30 mm diameter, 100 mm length), using decreasingly polar mixtures of water (containing 1% formic

acid) and MeCN as eluents (gradient 5-95%). Fractions containing the desired compound were evaporated to dryness to afford 2-(5-chloro-1-(piperidin-4-yl)-1H-indol-4-yl)-2,5-diazaspiro[3.4]octan-6-one (4 mg, 20%) as a white solid.

LCMS: m/z calcd. for [M+H$^+$] 359.2, found: 359.1; RT = 1.06 min.

^{1}H NMR (500 MHz, MeOD) δ ppm: 8.55 (s, 1H, NH), 7.22 (d, *J* = 3.5 Hz, 1H), 7.00 (d, *J* = 8.7 Hz, 1H), 6.93 (d, *J* = 8.7 Hz, 1H), 6.68 (d, *J* = 3.5 Hz, 1H), 4.60 – 4.56 (m, 2H), 4.52 (tt, *J* = 11.8, 4.0 Hz, 1H), 4.47 – 4.41 (m, 2H), 3.47 – 3.40 (m, 2H), 3.11 (td, *J* = 13.0, 2.9 Hz, 2H), 2.55 – 2.48 (m, 2H), 2.47 – 2.37 (m, 2H), 2.20 – 2.14 (m, 2H), 2.13 – 2.00 (m, 2H).

7-(1-cyclohexylbenzimidazol-2-yl)-1-methyl-pyrido[2,3-b][1,4]oxazin-2-one (compound S1)

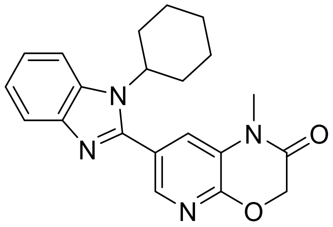

1-Methyl-2-oxo-pyrido[2,3-b][1,4]oxazine-7-carboxylic acid (19 mg, 0.0913 mmol), N2-cyclohexylbenzene-1,2-diamine (22.58 mg, 0.119 mmol), HATU (41.65 mg, 0.11 mmol), and triethylamine (50.6 μL, 0.365 mmol) were dissolved in dimethylformamide (0.5 mL) at room temperature. The reaction mixture was stirred for 5 minutes, followed by the addition of acetic acid (1 mL), and further stirred for 17 hours at room temperature. The reaction progress was monitored by HPLC-MS, confirming complete conversion to the desired product. The reaction mixture was diluted with methanol and water, filtered, and purified by semipreparative HPLC. Suitable fractions were combined and evaporated under reduced pressure which afforded 7-(1-cyclohexylbenzimidazol-2-yl)-1-methyl-pyrido[2,3-b][1,4]oxazin-2-one (12.3 mg, 37% yield).

^{1}H NMR (400 MHz, DMSO-d6) δ ppm: 8.23 (d, *J* = 2.0 Hz, 1H), 8.12-8.18 (m, 1H), 7.81-7.87 (m, 2H), 7.48-7.54 (m, 2H), 5.04 (s, 2H), 4.38 (tt, *J* = 12.3, 3.8 Hz, 2H), 2.19-2.44 (m, 2H), 2.05 (d, *J* = 10.6 Hz, 2H), 1.86 (d, *J* = 11.7 Hz, 2H), 1.61-1.68 (m, 1H), 1.30-1.47 (m, 3H).

7-(1-isopropyl-1H-benzo[d]imidazol-2-yl)-1H-pyrido[2,3-b][1,4]oxazin-2(3H)-one (compound 13)

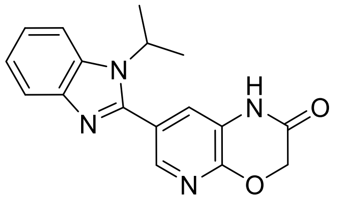

2-oxo-1H,2H,3H-pyrido[2,3-b][1,4]oxazine-7-carboxylic acid (19.8 mg, 0.1 mmol) and N,N-diisopropylethylamine (DIPEA, 51.6 μL, 0.3 mmol) were dissolved in dimethylformamide (2 mL) at room temperature. The mixture was stirred briefly, followed by the addition of HATU (41.8 mg, 0.11 mmol) and N1-(propan-2-yl)benzene-1,2-diamine (15.0 mg, 0.1 mmol). The reaction was stirred for 1 hour at room temperature. LC-MS analysis confirmed the formation of the intermediate product. The reaction mixture was filtered through basic alumina, washed with DMF/MeOH (9:1), and the solvent was removed under reduced pressure. To achieve cyclization, the residue was treated with acetic acid (2 mL) and heated under reflux conditions

for 16 hours. LC-MS analysis confirmed the formation of the desired product. The reaction mixture was concentrated, dissolved in DMF, and purified using preparative LC-MS. The purified fractions were lyophilized to afford the product 1H,2H,3H-pyrido[2,3-b][1,4]oxazin-2-one (25.1 mg, 81.4 mmol) as a yellow resin.

^{1}H NMR (400 MHz, DMSO-d6) δ ppm: 11.17 (s, 1H), 8.17 (d, J = 2.0 Hz, 1H), 8.03-8.11 (m, 1H), 7.75-7.88 (m, 1H), 7.58 (d, J = 2.2 Hz, 1H), 7.48 (dd, J = 6.1, 3.2 Hz, 2H), 4.94 (s, 2H), 4.78 (spt, J = 13.8 Hz, 1H), 1.66 (d, J = 6.8 Hz, 6H).

Data Tables

Table S1. SPR Data Table CRBN^{midi}

Affinity of CRBN binders measured by SPR C-avi CRBN^{midi}-on-chip, using a steady state affinity model.

Compound	Compound Class	Kd (nM)					Assay top concentration (nM)
		1	2	3	Mean	SD	
Lenalidomide	IMiD	135	371	379	295.0	138.6	500
Compound **9**	DHU	97.3	107	119	107.8	10.9	500
Compound **10**	Cyclimid	32100	33800	32300	32733	929.16	100000
Compound **11**	Phenylglutarimide	2560	2470	2100	2376.7	243.8	10000
Compound **12**	Aminoglutarimide	2530	2510	2520	2520.0	10	100000
Compound **8**	5,4-Spiro	187	194	183	188.0	5.57	1000
Compound **4**	Morpholinone	280	270	244	264.3	19.1	1000
Compound **S1**	Morpholinone, negative control	18100	19200	19300	18900	665.8	100000

Table S2. ITC measurements of CRBN binders with CRBN:DDB1$^{\Delta BPB}$

Compound	Compound Class	Measured Parameters				Classification
		K_D (μM)	N	ΔH (kJ/mol)	−TΔS (kJ/mol)	
Lenalidomide	IMiD	0.223 ± 0.025	1.78	10.8 ± 0.074	−48.8	exothermic
**4**	Morpholinone	0.899 ± 0.055	1.66	29.8 ± 0.186	−4.74	endothermic
**8**	5,4-Spiro	2.02 ± 0.102	1.24	−28.8 ± 0.238	−3.74	endothermic
**9**	DHU	0.039 ± 0.007	1.08	9.77 ± 0.123	−52.1	exothermic
**11**	Phenylglutarimide	8.51 ± 0.59	1.42	19.3 ± 0.405	−48.3	exothermic

Table S3. Measurement of different CRBN classes by DSF with $CRBN^{midi}$. Turbidity first derivative of thermal denaturation for $CRBN^{midi}$ in the absence or presence of binders.

Compound	Compound Class	$CRBN^{midi}$ Tm, turbidity (°C)						$CRBN^{TBD}$
		1	2	3	Mean	SD	Average Δ Tm	Average Δ Tm
Apo	/	44.07	44.13	44.05	44.08	0.04	/	/
Lenalidomide	IMiD	58.03	58.04	58.11	58.05	0.05	13.97	3.6
**4**	Morpholinone	41.15	41.16	41.21	41.17	0.03	-2.91	7.4
**8**	5,4-Spiro	47.18	47.38	47.29	47.29	0.10	3.21	6.1
**9**	Dihydrouracil	58.61	58.65	58.67	58.65	0.03	14.57	5.1
**10**	Cyclimid	55.00	54.92	54.95	54.96	0.04	10.88	0.6
**11**	Phenylglutarimide	55.96	56.14	56.01	56.03	0.09	11.95	2.6
**12**	Aminoglutarimide	55.47	55.58	55.41	55.49	0.08	11.41	n.d.

Table S4. Turbidity first derivative of thermal denaturation for $CRBN^{midi}$ WT and mutants in the absence or presence of binders. WT is mean of triplicates.

Mutation	Compound	Compound class	Tm	ΔTm
WT	Apo	/	44.08	-
WT	Lenalidomide	Imid	58.05	13.97
WT	Compound 9	DHU	58.65	14.57
WT	Compound 8	5,4-Spiro	47.29	3.21
WT	Compound 4	Morpholinone	41.17	-2.91
H378N	Apo	/	43.00	-
H378N	Lenalidomide	Imid	55.49	12.49
H378N	Compound 9	DHU	56.14	13.14
H378N	Compound 8	5,4-Spiro	44.70	1.70
H378N	Compound 4	Morpholinone	41.65	-1.35
H378A	Apo	/	43.05	-
H378A	Lenalidomide	Imid	53.99	10.94
H378A	Compound 9	DHU	53.91	10.86
H378A	Compound 8	5,4-Spiro	47.65	4.6
H378A	Compound 4	Morpholinone	41.81	-1.24

Mutation	Compound	Compound class	Tm	ΔTm
Q100A	Apo	/	44.00	-
Q100A	Lenalidomide	Imid	58.00	14.00
Q100A	Compound 9	DHU	58.75	14.75
Q100A	Compound 8	5,4-Spiro	47.17	3.17
Q100A	Compound 4	Morpholinone	41.37	-2.63
L60A	Apo	/	44.31	-
L60A	Lenalidomide	Imid	51.38	7.07
L60A	Compound 9	DHU	49.53	5.22
L60A	Compound 8	5,4-Spiro	46.47	2.16
L60A	Compound 4	Morpholinone	41.62	-2.69
L60A H378A	Apo	/	42.80	-
L60A H378A	Lenalidomide	Imid	49.23	6.43
L60A H378A	Compound 9	DHU	47.45	4.65
L60A H378A	Compound 8	5,4-Spiro	43.79	0.99
L60A H378A	Compound 4	Morpholinone	39.83	-2.97

Table S5. Small Angle X-ray scattering statistics for CRBN^{midi} bound to each binder series

SASBDB ID	X	X	X	X	X	X
**Sample Details**						
Organism	*E. coli* BL21 (DE3)					
Source	Recombinantly expressed					
Uniprot sequence ID	*Derived from human Cereblon, Uniprot Q96SW2*					
Description	CRBN^{midi}	CRBN^{midi} bound to lenalidomide	CRBN^{midi} bound to Compound **9**	CRBN^{midi} bound to Compound **11**	CRBN^{midi} bound to Compound **8**	CRBN^{midi} bound to Compound **4**
Expected molecular mass (kDa)	37.4	37.7	37.7	37.7	37.7	37.7
Loading concentration (mg/mL)	3.4					
Injection volume (µL)	50					
Buffer composition	20 mM HEPES pH 7.5, 500 mM NaCl, 0.5 mM TCEP					
**SAS data collection parameters**						
Source and instrument	B21, Diamond Light Source					
Wavelength (Å)	0.9464					
Sample-detector distance (m)	3.7					
q-measurement range (Å)	4.5×10^{-3}- 3.4×10^{-1}					
Radiation damage monitoring	Frame-by-frame comparison					
Exposure time (s) and number	3 x 600					
Sample configuration	SEC-SAXS, Cytiva Superdex 200 Increase 3.2/300					
Sample temperature (°C)	15					
**Structural parameters**						
Guinier analysis						
I(0) (cm^{-1})	$0.017 \pm 3.4 \times 10^{-5}$	$0.017 \pm 2.4 \times 10^{-5}$	$0.017 \pm 2.8 \times 10^{-5}$	$0.012 \pm 1.6 \times 10^{-5}$	$0.021 \pm 2.6 \times 10^{-5}$	$0.0096 \pm 2.4 \times 10^{-5}$
R_g (Å)	26.83 ± 0.09	22.99 ± 0.06	23.43 ± 0.07	22.80 ± 0.06	26.04 ± 0.06	25.72 ± 0.11
qR_g max	1.28	1.30	1.30	1.30	1.30	1.30
P(r) analysis from AUTOGNOM						
I(0) (cm^{-1})	$0.016 \pm 2.7 \times 10^{-5}$	$0.017 \pm 1.9 \times 10^{-5}$	$0.017 \pm 2.8 \times 10^{-5}$	$0.012 \pm 1.5 \times 10^{-5}$	$0.021 \pm 2.5 \times 10^{-5}$	$0.0095 \pm 2.2 \times 10^{-5}$
R_g (Å)	26.26 ± 0.051	22.56 ± 0.035	23.11 ± 0.054	22.55 ± 0.046	26.05 ± 0.0049	25.43 ± 0.060
d_{max} (Å)	154.26	137.92	141.94	141.42	161.52	145.16
q range (Å^{-1})	0.0126-0.2982	0.0089-0.3400	0.0093-0.3400	0.0111-0.3400	0.0101-0.3071	0.0126-0.3110
χ^2 (total estimate from *GNOM*)	0.87	0.86	0.81	0.78	0.83	0.85

Porod volume (Å^{-3}) (ratio V_p/calculated M)	70306.90	59262.70	61505.60	63949	60447.10	55533.10

Table S6. Small Angle X-ray Scattering statistics for CRBN^{midi} mutants bound to compound 9

SASBDB ID	X	X	X	X	X
			Sample Details		
Organism			*E. coli* BL21 (DE3)		
Source			Recombinantly expressed		
Uniprot sequence ID			*Derived from human Cereblon, Uniprot Q96SW2*		
Description	CRBN^{midi} H378A	CRBN^{midi} H378N	CRBN^{midi} L60A	CRBN^{midi} L60A H378A	CRBN^{midi} Q100A
Expected molecular mass (kDa)	37.4	37.7	37.7	37.6	37.7
Loading concentration (mg/mL)			3.4		
Injection volume (µL)			50		
Buffer composition			20 mM HEPES pH 7.5, 500 mM NaCl, 0.5 mM TCEP		
			SAS data collection parameters		
Source and instrument			B21, Diamond Light Source		
Wavelength (Å)			0.9464		
Sample- detector distance (m)			3.7		
q-measurement range (Å)			4.5×10^{-3}- 3.4×10^{-1}		
Radiation damage monitoring			Frame-by-frame comparison		
Exposure time (s) and number			3 x 600		
Sample configuration			SEC-SAXS, Cytiva Superdex 200 Increase 3.2/300		
Sample temperature (°C)			15		
			Structural parameters		
			Guinier analysis		
I(0) (cm^{-1})	$0.012 \pm 1.6 \times 10^{-5}$	$0.013 \pm 2.9 \times 10^{-5}$	$0.012 \pm 1.8 \times 10^{-5}$	$0.014 \pm 2.0 \times 10^{-5}$	$0.011 \pm 1.7 \times 10^{-5}$
R_g (Å)	22.88 ± 0.06	23.80 ± 0.10	24.84 ± 0.07	25.18 ± 0.06	23.07 ± 0.07
qR_g max	1.30	1.30	1.29	1.30	1.30
			P(r) analysis from AUTOGNOM		
I(0) (cm^{-1})	$0.011 \pm 1.5 \times 10^{-5}$	$0.012 \pm 2.3 \times 10^{-5}$	$0.012 \pm 1.8 \times 10^{-5}$	$0.014 \pm 1.7 \times 10^{-5}$	$0.011 \pm 1.3 \times 10^{-5}$
R_g (Å)	22.35 ± 0.033	22.83 ± 0.041	24.56 ± 0.054	25.05 ± 0.039	22.38 ± 0.032
d_{max} (Å)	124.76	123.82	145.48	149.88	125.78
q range (Å^{-1})	0.0083-0.3400	0.0091-0.3361	0.0121-0.3220	0.0090-0.3177	0.0082-0.3400
χ^2 (total estimate from *GNOM*)	0.87	0.85	0.82	0.86	0.86
Porod volume (Å^{-3}) (ratio V_p/calculated M)	54576	64098	61883	63723	56333

Table S7. X-Ray Crystallographic Data and Refinement Statistics Data

	CRBN^{midi}:Compound **9**
**Data collection**	
Space group	$P12_11$
Cell dimensions	
a, *b*, *c* (Å)	51.42, 153.11, 54.05
α, β, γ (°)	90.00, 118.40, 90.00
Resolution (Å)	47.54-2.36 (2.56-2.36)
R_{merge}	0.159 (1.459)
I / s*I*	7 (1.6)
$CC_{1/2}$	0.994 (0.657)
Completeness (%) (spherical)	59.2 (13.6)
Completeness (%) (ellipsoidal)	87.1 (70.7)
Redundancy	6.3 (5.8)
**Refinement**	
Resolution (Å)	47.54-2.36
No. reflections	17700 (179)
R_{work} / R_{free}	0.233/ 0.296
No. atoms	5041
Protein	4978
Ligand/ion	48
Water	15
B-factors	
Protein	47.11
Ligand/ion	37.74
Water	35.31
R.m.s. deviations	
Bond lengths (Å)	0.018
Bond angles (°)	0.49
**PDB ID**	9SUN

Table S8. Cryo-EM data collection, processing and refinement statistics for dihydrouracil **9**

	Cryo-EM structure of CRBN bound to 1-[1-(4-piperidyl)indol-4-yl]hexahydropyrimidine-2,4-dione in the closed conformation			Cryo-EM structure of CRBN bound to yl]hexahydropyrimidine-2,4-dione in th	
	Composite map EMD-55258 PDB 9SVH	Consensus map EMD-55057	Focused map EMD-55058	Composite map EMD-55259 PDB 9SVI	Consensus map EMD-55059
**Data collection and processing**					
Magnification	190,000				
Voltage (kV)	200				
Electron exposure (e–/Å^2)	21.94				
Defocus range (μm)	-(1.7-3.2)				
Pixel size (Å)	0.71				
Symmetry imposed	C1				
Initial particle images (no.)	1,585,848				
Final particle images (no.)		56,510			86,485
Map resolution (Å)		3.66	4.07		3.33
FSC threshold		0.143	0.143		0.143
**Refinement**					
Map sharpening *B* factor (Å^2)		144.2	209.9		141.6
Model composition					
Non-hydrogen atoms	9,164			8,846	
Protein residues	1,174			1,183	
Ligands	A1JP8, ZN			A1JP8, ZN	
Model-Map					
CC (mask)	0.69			0.76	
R.m.s. deviations					
Bond lengths (Å)	0.004			0.003	
Bond angles (°)	0.630			0.622	
Validation					
MolProbity score	1.65			1.45	
Clashscore	6.29			4.20	
CaBLAM outliers (%)	1.48			1.89	
Rotamer outliers (%)	0.00			0.00	
Cβ outliers (%)	0.00			0.00	
Ramachandran plot					
Favored (%)	95.69			96.26	
Allowed (%)	4.31			3.66	
Disallowed (%)	0.00			0.09	

Table S9. Cryo-EM data collection, processing and refinement statistics for spirolactam **8**

	Cryo-EM structure of CRBN bound to 2-[5-chloro-1-(4-piperidyl)indol-4-yl]-2,5-diazaspiro[3.4]octan-6-one in the open conformation		
	Composite map<br>EMD-55257<br>PDB 9SVG	Consensus map<br>EMD-54624	Focused map<br>EMD-54644
**Data collection and processing**			
Magnification		165,000	
Voltage (kV)		300	
Electron exposure (e–/Å^2)		50	
Defocus range (μm)		-(0.8-1.8)	
Pixel size (Å)		0.75	
Symmetry imposed		C1	
Initial particle images (no.)		2,575,103	
Final particle images (no.)		573,499	
Map resolution (Å)		2.22	3.44
FSC threshold		0.143	0.143
**Refinement**			
Map sharpening *B* factor (Å^2)		69	145.1
Model composition			
Non-hydrogen atoms	8,880		
Protein residues	1158		
Ligands	A1JQZ, ZN		
Model-Map			
CC (mask)	0.67		
R.m.s. deviations			
Bond lengths (Å)	0.003		
Bond angles (°)	0.681		
Validation			
MolProbity score	1.46		
Clashscore	4.78		
CaBLAM outliers (%)	2.12		
Rotamer outliers (%)	0.11		
Cβ outliers (%)	0.00		
Ramachandran plot			
Favored (%)	96.68		
Allowed (%)	3.23		
Disallowed (%)	0.09		

Table S10. List of primers for sire-directed mutagenesis

Primer Name	Sequence 5'-3'	Mutation introduced
L60A	CCGACATCACACACCTACGCGGGTGCCGATATGGAAG	Mutate Leu60 to Ala in $CRBN^{midi}$
Q100A	CAGACCTTACCGCTGGCGCTGTTTCACCCGCAGGAAG	Mutate Gln100 to Ala in $CRBN^{midi}$
H378A	CGCCCGTCTACCGAAGCCAGCTGGTTTCCAGGGTATG	Mutate His378 to Ala in $CRBN^{midi}$ and $CRBN^{midiL60A}$
H378N	CGCCCGTCTACCGAAAACAGCTGGTTTCCAGGGTATG	Mutate His378 to Asn in $CRBN^{midi}$

Table S11. List of cycling parameters for site-directed mutagenesis

Segment	Cycles	Temperature (°C)	Time (minutes)
1	1	95	1
2	15	95	0.5
		55	1
		68	8.5

Table S12. Intact protein LC-MS of $CRBN^{midi}$ mutants

Mutant	Predicted Mass (Da)	Experimental Mass (Da)
WT	37435.88	37428.77
H378N	37412.84	37406.20
H378A	37369.82	37362.47
Q100A	37378.83	37370.89
L60A	37393.80	37386.22
L60A H378A	37327.74	37318.07

Table S13. Primers for cloning CRBN:$DDB1^{\Delta BPB}$

Primer Name	Sequence
DDB1delBPBvec_F	5'GGAAACGGTAACTCTGGAGAGATCCAGAAGCTGCACATCCGTACCGTGCCTC 3'
DDB1delBPBvec_R	3'GCTTTTGAGCGGTGACCACGTAGTTGTAGGA 5'
DDB1delBPBins_F	5'TCCTACAACTACGTGGTCACCGCTCAAAAGC 3'
DDB1delBPBins_R	3'TCCAGAGTTACCGTTTCCACCGATACCGTTACGGATGATACGCAGGG 5'

Supplementary Figures

Figure S1. 2D ^{15}N HSQC CSP detected with thalidomide and 15N-labelled CRBN^{TBD}

Chemical shift perturbation and line broadening induced upon binding of thalidomide to CRBN TBD. Circles indicate cross peaks affected by the FBS hit depicted in Fig. 2 suggesting that both compounds interact with the same binding site

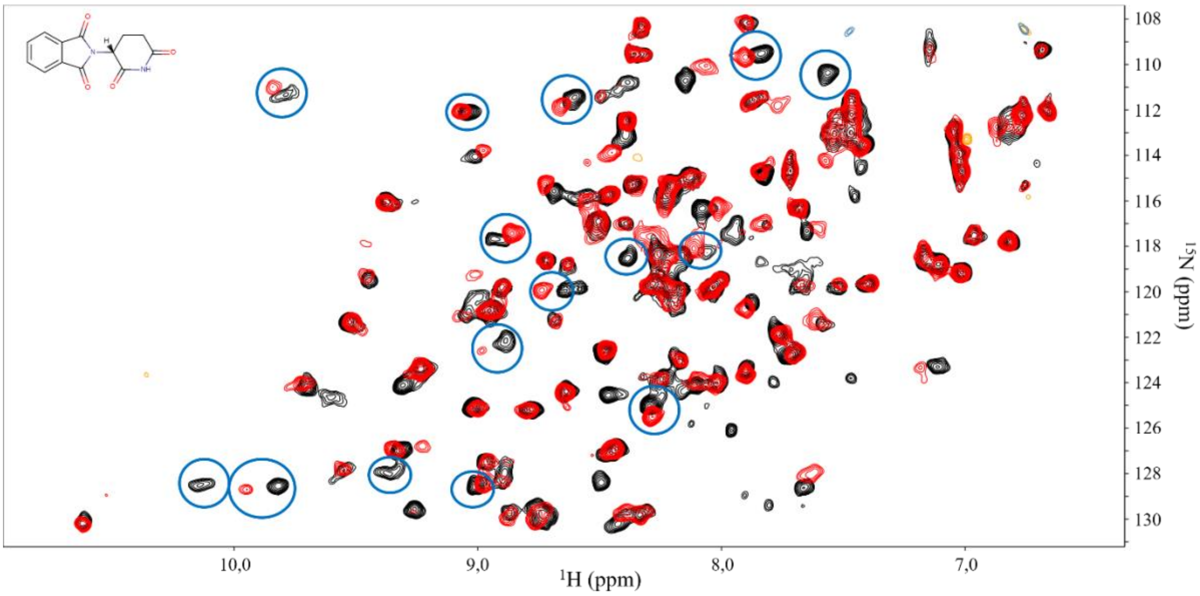

Figure S2. CRBN^{midi}:Dihydrouracil **9** crystal structure domain swap and comparison to other structures.

Structure of CRBN^{midi} in complex with **9**. CRBN^{midi} is shown in cartoon representation coloured by domain, Lon (yellow), HB (orange), TBD (salmon), GSG linker (cyan), **9** is shown as black sticks. A) The left panel shows the structure with the domain swap modelled and the right panel shows the structure without the domain swap modelled. The GSG linker is shown in stick form and coloured cyan, the polder OMIT (Fo-Fc) map of the linker contoured to 3 σ is shown as green mesh. B) Superposition of TBD (chain A) of CRBN^{midi}:**9** with the crystal structure of CRBN^{TBD}:compound **C3** (grey, PDB ID: 9ODS). Chemical structures of compound **C3** and DHU **9** are show below.

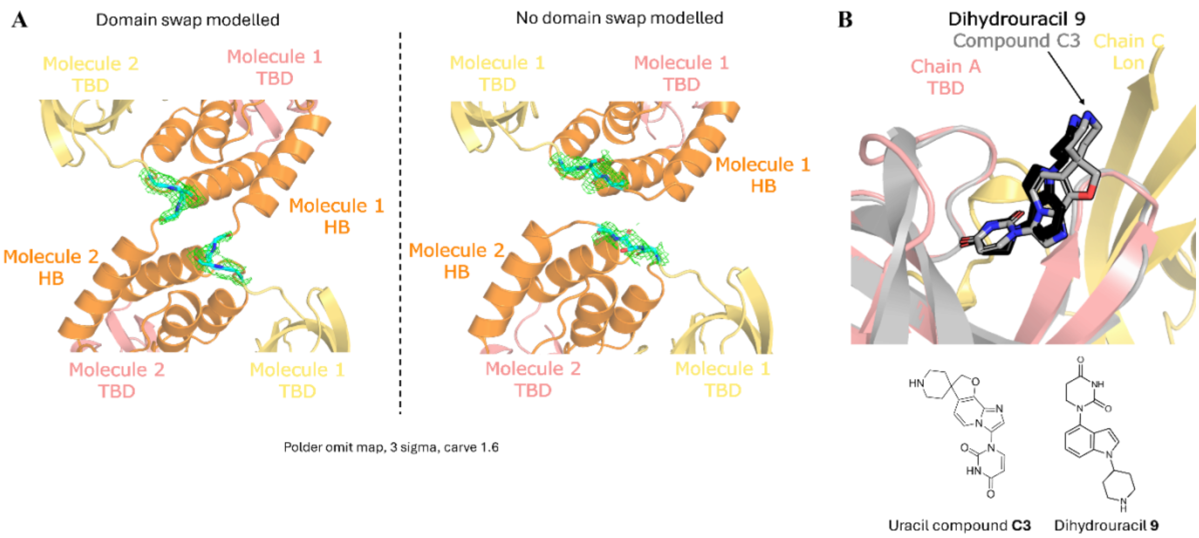

Figure S3. Cryo-EM Workflow for dihydrouracil **9**

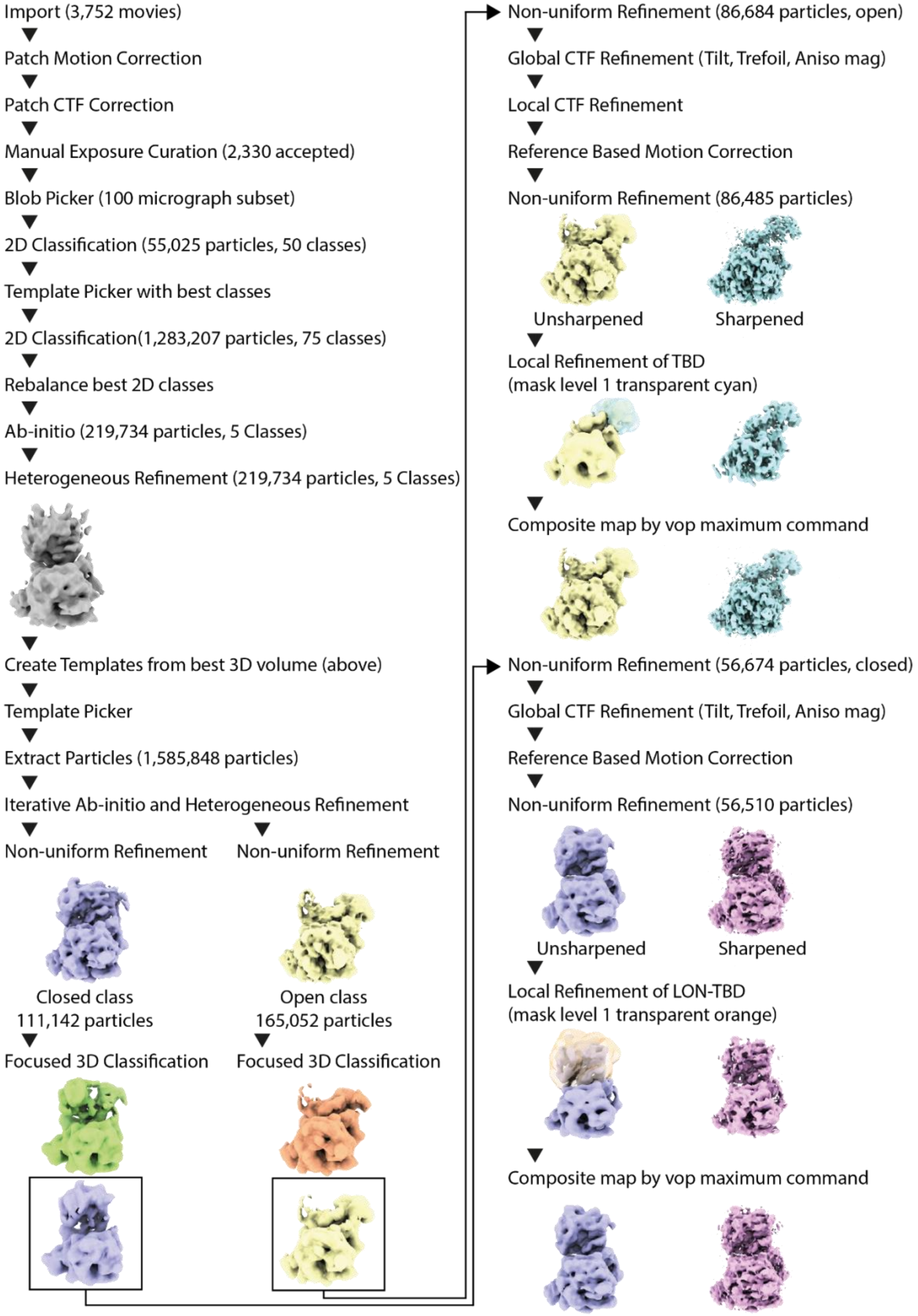

Figure S4. Cryo-EM Map Information for dihydrouracil **9**

Workflow for Cryo-EM data processing of dataset for CRBN-DDB1ΔBPB bound to dihydrouracil ligand 1-[1-(4-piperidyl)indol-4-yl]hexahydropyrimidine-2,4-dione. Gold-standard Fourier shell correlation, cFSC, angular distribution, and posterior position directional distribution plots for the consensus and locally refined maps of CRBN-DDB1ΔBPB bound to dihydrouracil ligand 1-[1-(4-piperidyl)indol-4-yl]hexahydropyrimidine-2,4-dione in the open and closed conformation.

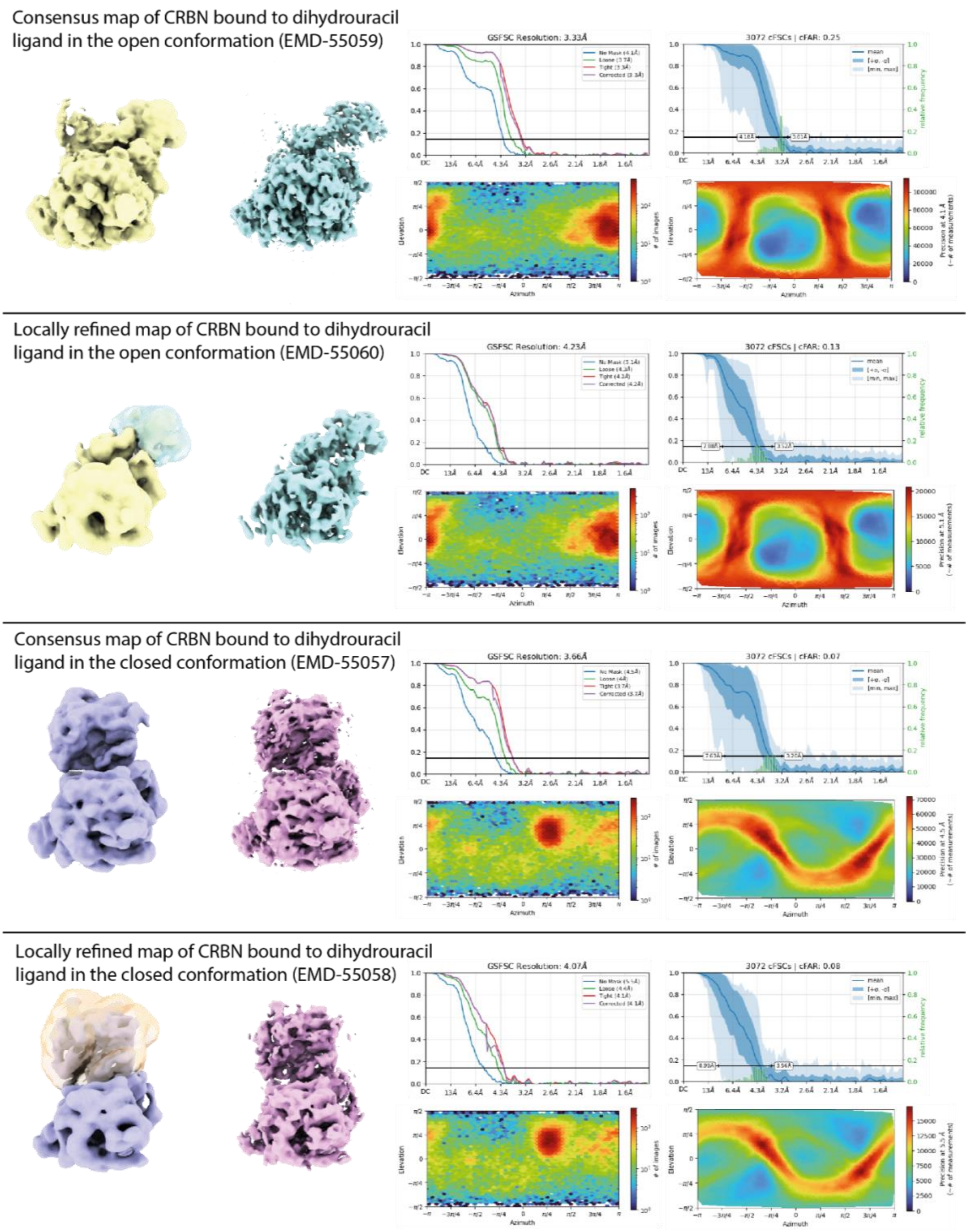

Figure S5. Cryo-EM Workflow for 5,4-spiro **8**

Workflow for Cryo-EM data processing of dataset for CRBN-DDB1ΔBPB bound to spirocyclic ligand 2-[5-chloro-1-(4-piperidyl)indol-4-yl]-2,5-diazaspiro[3.4]octan-6-one2-[5-chloro-1-(4-piperidyl)indol-4-yl]-2,5-diazaspiro[3.4]octan-6-one.

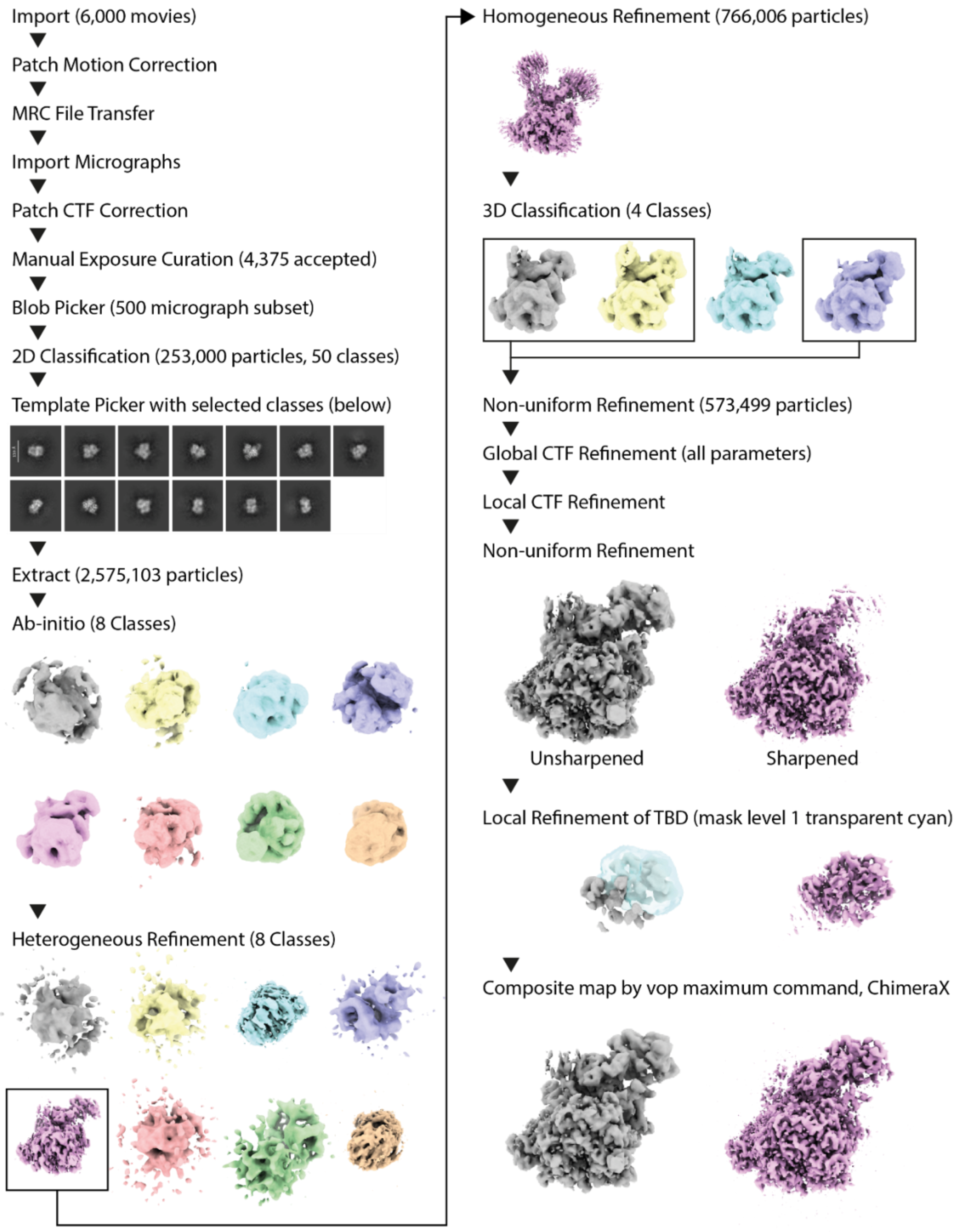

Figure S6. Cryo-EM Map Information for 5,4-spiro **8**

Gold-standard Fourier shell correlation, cFSC, angular distribution, and posterior position directional distribution plots for the consensus and locally refined maps of CRBN-DDB1ΔBPB bound to spirocyclic ligand 2-[5-chloro-1-(4-piperidyl)indol-4-yl]-2,5-diazaspiro[3.4]octan-6-one2-[5-chloro-1-(4-piperidyl)indol-4-yl]-2,5-diazaspiro[3.4]octan-6-one.

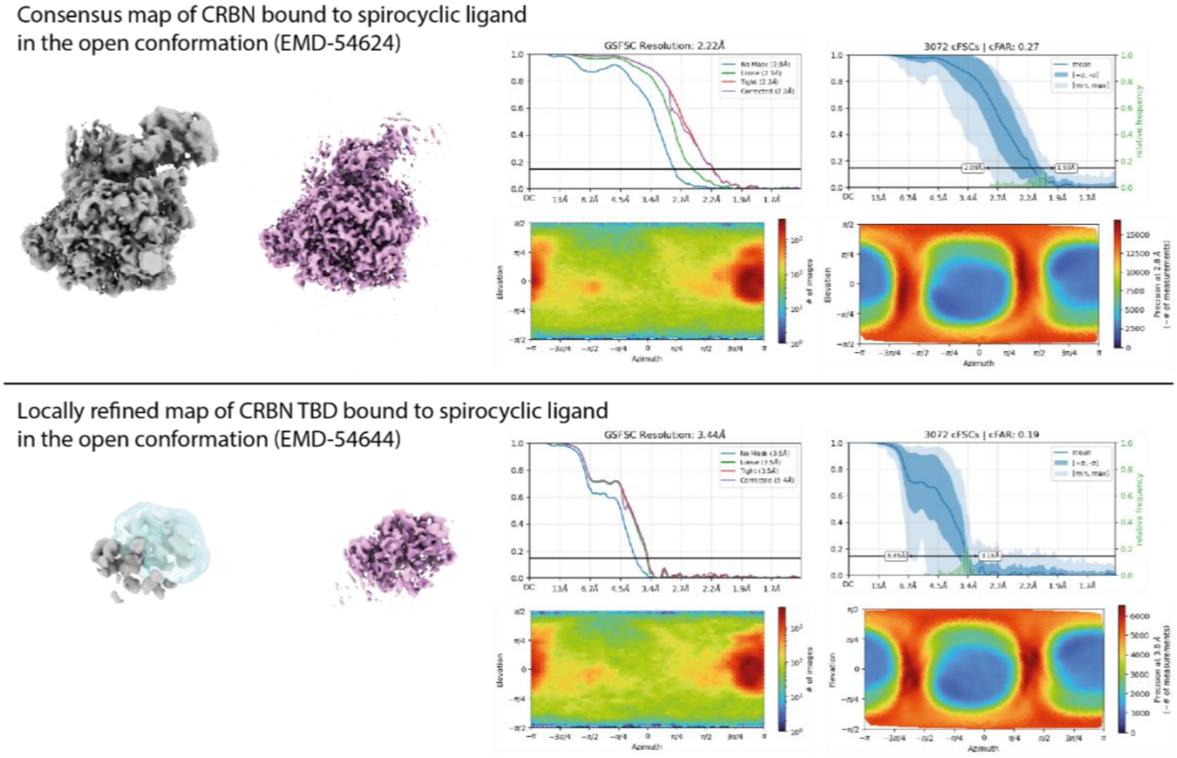

Figure S7. 5,4-spiro **8** Binding Site in Locally Refined Cryo-EM Map

Locally refined map of TBD bound to spirolactam 8 showing binding site.

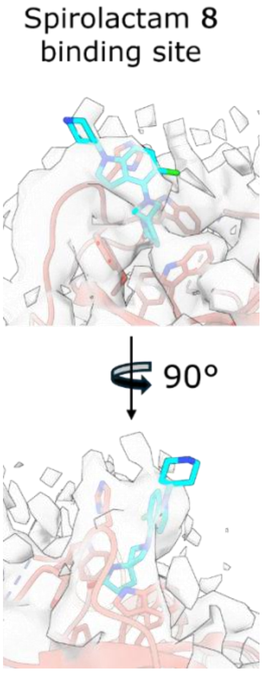

Figure S8. Validating the stability of FLAG-CRBN expressed into DLD-1 CRBN KO background cells.

A

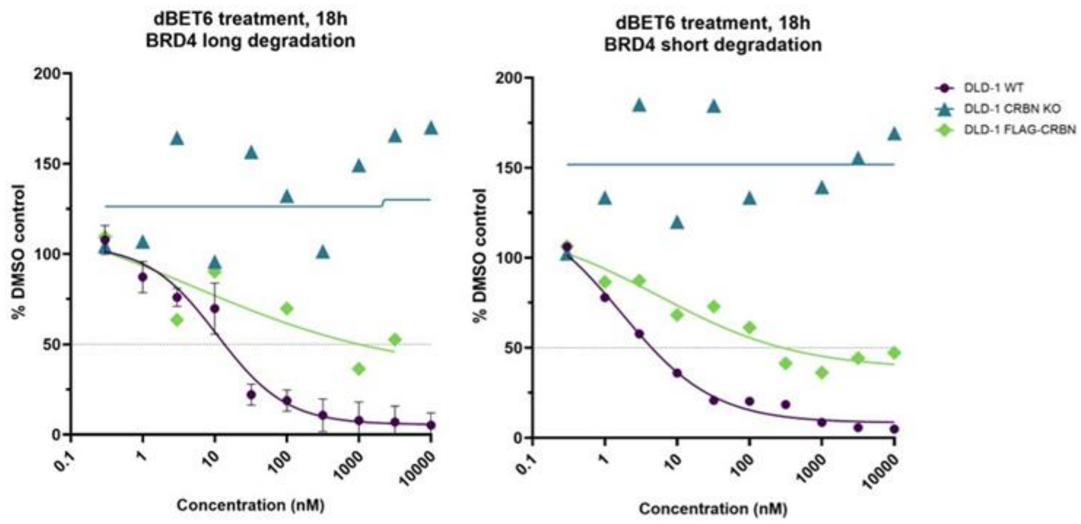

B

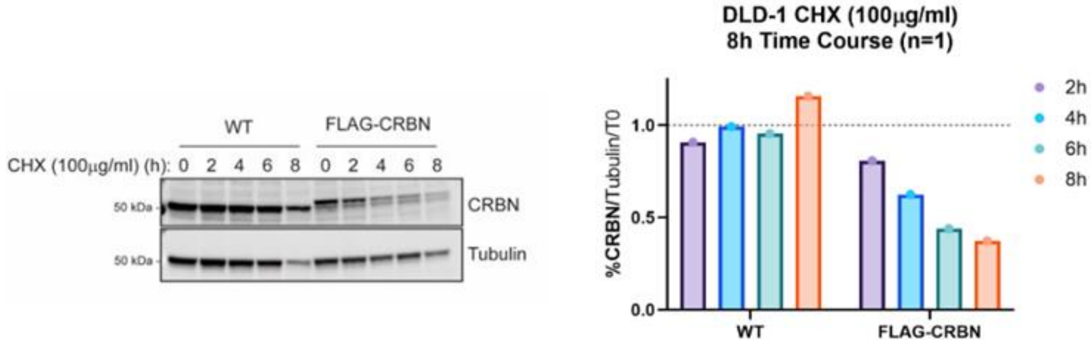

Cells were treated with 100 mg/ml cycloheximide (CHX) over an 8h time-course. CRBN levels were quantified and normalised against loading control and time zero (T0) expression.

Figure S9. BRD4 Degradation in DLD-1 WT, CRBN KO and CRBN KO stably expressing FLAG-CRBN upon Treatment with dBET6

Representative western blots showing degradation of BRD4 in (A) DLD-1 wild-type (WT), (B) CRBN-KO and (C) CRBN KO stably expressing FLAG-CRBN. Cells were treated with dBET6 for 18h before analysis by western blotting. Quantification of all replicates is summarised in Figure S8A.

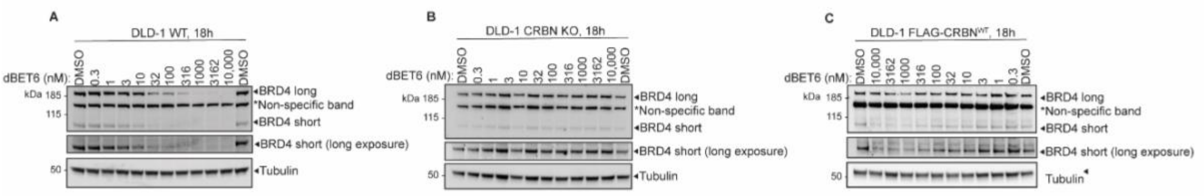

References

1. Ichikawa, S. *et al.* The cyclimids: Degron-inspired cereblon binders for targeted protein degradation. *Cell Chem Biol* **31**, 1162-1175.e10 (2024).
2. Min, J. *et al.* Phenyl-Glutarimides: Alternative Cereblon Binders for the Design of PROTACs. *Angewandte Chemie - International Edition* **60**, 26663–26670 (2021).
3. Lee, E. C. Y. & et al. SMARCA2 degraders and uses thereof WO2024256988A1. (2024).
4. Nasceschuk, C. G. & et al. Isoindolinone and indazole compounds for degradation of EGFR WO2021127561A1. (2021).
